# Decoding the Digits: How Number Notation Influences Cognitive Effort and Performance in Chinese-to-English Sight Translation

**DOI:** 10.3390/bs15091195

**Published:** 2025-09-01

**Authors:** Xueyan Zong, Lei Song, Shanshan Yang

**Affiliations:** 1 School of Foreign Languages, Central China Normal University, Wuhan 430079, China; 2 School of Fine Arts, Central China Normal University, Wuhan 430079, China

**Keywords:** number notation, sight translation, eye-tracking, cognitive effort, performance

## Abstract

Numbers present persistent challenges in interpreting, yet cognitive mechanisms underlying notation-specific processing remain underexplored. While eye-tracking studies in visually-assisted simultaneous interpreting have advanced number research, they predominantly examine Arabic numerals in non-Chinese contexts—neglecting notation diversity increasingly prevalent in computer-assisted interpreting systems where Automatic Speech Recognition outputs vary across languages. Addressing these gaps, this study investigated how number notation (Arabic digits vs. Chinese character numbers) affects trainee interpreters’ cognitive effort and performance in Chinese-to-English sight translation. Employing a mixed-methods design, we measured global (task-level) and local (number-specific) eye movements alongside expert assessments, output analysis, and subjective assessments. Results show that Chinese character numbers demand significantly greater cognitive effort than Arabic digits, evidenced by more and longer fixations, more extensive saccadic movements, and a larger eye-voice span. Concurrently, sight translation quality decreased markedly with Chinese character numbers, with more processing attempts yet lower accuracy and fluency. Subjective workload ratings confirmed higher mental, physical, and temporal demands in Task 2. These findings reveal an effort-quality paradox where greater cognitive investment in processing complex notations leads to poorer outcomes, and highlight the urgent need for notation-specific training strategies and adaptive technologies in multilingual communication.

## 1. Introduction

In conference interpreting, a cognitively challenging task requiring the simultaneous performance of several subtasks, interpreters must navigate myriad input variables that significantly influence their cognitive processing and performance outcomes (Christoffels & de Groot, 2005; Pöchhacker, 2022). Among these variables, numbers represent a particularly formidable challenge, capable of disrupting the interpreting flow and taxing even seasoned professionals (Gile, 2009; Frittella, 2019a, 2019b). The challenges associated with number interpreting stem from factors such as low predictability, limited contextual relevance, high information density, and reduced linguistic redundancy in speeches (Braun & Clarici, 1996; Mazza, 2001; Cheung, 2009; Morrato, 2011; Frittella, 2019a). As Mead (2015) highlights, the cognitive burden on interpreters during number processing is indeed substantial.

This challenge becomes even more pronounced in cross-linguistic contexts with fundamentally different numerical systems. While numerical cognition is universal, its lexical and structural encoding varies substantially across languages (Xu et al., 2020). For example, the structural mismatches between Chinese decimal-based numerical units (e.g., 10-thousand/十万-100 thousand) and English magnitude segmentation (e.g., thousand/million) require interpreters to perform real-time arithmetic conversions. Such conversions compete for finite cognitive resources, escalating cognitive load (Kahneman, 1973) and intensifying the likelihood of errors during bilingual processing (Campbell et al., 1999). Beyond these cross-linguistic structural differences, number notation itself constitutes a critical yet underexplored variable in cognitive processing. Chinese presents a unique case, with numbers appearing as either Arabic digits (e.g., “1”) or two forms of Chinese characters (simplified “一” or traditional “壹”). Research in numerical cognition suggests that these different notations engage distinct cognitive pathways, with potential implications for processing efficiency in interpreting contexts (Campbell et al., 1999; Cao et al., 2010a). This evidence highlights the importance of examining notation effects in interpreting contexts, particularly for languages like Chinese where multiple numerical formats coexist. However, these notation-specific effects, despite their theoretical significance and practical relevance, have received minimal attention in interpreting studies.

Recent technological advancements, particularly real-time Automatic Speech Recognition (ASR), have transformed conference interpreting by introducing a visual dimension to this traditionally auditory-dominant activity. This evolution toward computer-assisted interpreting (CAI) has created both opportunities and challenges for number processing. Eye-tracking research shows interpreters strategically allocate visual attention to numerical content (Korpal & Stachowiak-Szymczak, 2018) with resulting accuracy improvements. Simultaneously, technology introduces new cognitive challenges such as the diversity and inconsistency of number notations in ASR outputs (Defrancq & Fantinuoli, 2021) and information overload on visual displays (Seubert, 2019). These developments highlight an emerging research imperative: understanding how different number presentation formats influence cognitive processing in increasingly multimodal interpreting environments.

A critical limitation in current research is the almost exclusive focus on Arabic numerals in European language pairs—overlooking the Chinese numerical system, which Mead (2015, p. 287) identifies as presenting “even more pronounced” difficulties for interpreters. To address this critical gap, our study employs eye-tracking technology to investigate how different number notations (Arabic digits versus Chinese characters) influence cognitive processing and performance during Chinese-to-English sight translation. As visual numerical displays become increasingly common in professional settings (Defrancq & Fantinuoli, 2021), this research not only advances our understanding of notation-specific effects but also offers practical implications for technology-enhanced interpreting environments and interpreter training programs in today’s increasingly multimodal professional landscape.

## 2. Literature Review

Numbers represent a significant challenge for interpreters (Mead, 2015; Frittella, 2019a, 2019b), as they require the simultaneous processing of linguistic, mathematical, and cross-modal information. This complexity is further compounded by the need to convert between numeral systems in different languages, which often involves both auditory and visual modalities—a process termed “numerical transcoding” that typically requires interpreters to mentally convert heard number words into visual representations (such as Arabic numerals) before rendering them in the target language (Frittella, 2019a, p. 80). This cross-modal conversion places additional cognitive demands on interpreters.

Difficulties with number processing in interpreting are well documented by empirical research, which consistently shows that numerical content is a critical locus of errors and cognitive strain across interpreting modes and language pairs. For example, Alessandrini (1990) reported that the error rate for numbers was one-third greater than that for nonnumerical content in English-to-Italian consecutive interpreting, even among professional interpreters. Similarly, Braun and Clarici (1996) highlighted that student interpreters working from German into Italian made errors in 70% of number renditions during simultaneous interpreting. Mazza (2001) reported only a 53.9% accuracy rate for student interpreters processing numbers in English-to-Italian simultaneous interpreting, while Pinochi (2010) observed a 40% number error rate among student interpreters in German/English-to-Italian simultaneous interpreting. Recently, corpus-based analyses by Kajzer-Wietrzny et al. (2024) have corroborated these findings, using the EPTIC corpus of professional simultaneous interpreting across several European languages to reveal how numerical content affects not only accuracy but also delivery fluency. Such consistent findings underline that number processing remains one of interpreting’s most persistent and quantifiable difficulties.

Recent technological advancements have brought attention to how Automatic Speech Recognition (ASR) systems can alleviate cognitive burdens of processing numbers in interpreting practice. Studies have demonstrated that by converting verbal numbers into visually accessible forms, such as Arabic digits, these tools significantly improve interpreting accuracy. Desmet et al. (2018), for example, demonstrated that ASR conversion of number words to Arabic digits improved students’ number interpreting accuracy from 56.5% to 86.5%. Similarly, the use of tools like InterpretBank’s ASR module enabled trainee interpreters to reduce number processing errors from 39.8% to 14.8% (Pisani & Fantinuoli, 2021). Defrancq and Fantinuoli (2021) further confirmed these findings, showing that ASR-supported interpreting increased complete number renditions from 67.7% to 90.2%, with especially pronounced gains for complex or decimal numbers—where accuracy rose from 54.8% to 90.5%. Even traditional forms of visual support such as slide presentations have been shown to reduce the cognitive burden of number processing for both professional and trainee interpreters (Stachowiak-Szymczak & Korpal, 2019).

However, these benefits come with trade-offs. While technological tools demonstrably improve accuracy, they also introduce new cognitive complexities by shifting from purely auditory number processing to a multimodal input environment. This multimodal processing challenge is particularly well-documented in signed language interpreting where practitioners navigate between visual–gestural and auditory–vocal modalities, constantly managing competing attentional claims across distinct representational systems (Pöchhacker, 2021; Treviño et al., 2022). This multimodal complexity also aligns precisely with Gile’s (2009) Effort Model and Tightrope Hypothesis: visual ASR processing constitutes an additional cognitive “effort” that directly competes with listening, memory, and production efforts. Similarly, Pashler’s (1989) work on attentional bottlenecks identifies dual cognitive costs—perceptual interference and response selection constraints—when managing concurrent tasks across different modalities that compete for the same central processing mechanisms.

Empirical evidence substantiates this competition of cognitive resources. Yang (2023) noticed that interpreters’ visual attention to numbers was significantly longer than for other contextual elements. Sun et al. (2024) documented heightened visual processing demands when interpreters encountered number-dense ASR-generated subtitle segments, evidenced by increased fixation counts and durations that reflect greater cognitive investment in processing visual numerical information. The cognitive strain became particularly evident under increased temporal pressure. Korpal and Stachowiak-Szymczak (2020) discovered that faster delivery rates significantly increase interpreters’ fixation frequency on numbers displayed on slides, suggesting compensatory “scanning” pattern under cognitive strain. Experience differences are also apparent—Stachowiak-Szymczak and Korpal (2019) demonstrated that while visual slides significantly enhance numerical accuracy, they entail substantially higher cognitive costs for less experienced interpreters—manifested in prolonged visual engagement compared to professionals. Both objective measures and subjective reports from trainees (Desmet et al., 2018; Pisani & Fantinuoli, 2021) indicated distractions and heightened cognitive demands when reading ASR transcriptions during interpreting. Collectively, these findings suggest that technology redistributes rather than eliminates cognitive burdens—creating new competition between visual processing and articulatory processes.

An important but often overlooked limitation of current CAI interface design lies in the inconsistent formatting of numerical displays, which fundamentally affects cognitive processing efficiency. Defrancq and Fantinuoli (2021) observed that inconsistent number format representation in ASR output, for example, mixing written words “two” and digits “2”, can increase processing demands. Similarly, Pisani and Fantinuoli (2021) documented that ASR systems are prone to transcription errors (e.g., misrecognizing “2000” as “2006”) and unstable number format representation, which may further distract interpreters and compromise accuracy. J. Liu et al. (2024) further validated these concerns in their multilingual Computer-Assisted Interpreting (CAI) system research, demonstrating that while their system using ASR services from Tencent (one of China’s largest technology providers in AI and cloud services) successfully recognized numbers, it struggled with consistent formatting—displaying some as Arabic numerals and others as word forms. These findings illuminate a critical issue: while CAI systems aim to enhance performance through visual representation of numbers, the variability in how these numbers appear introduces its own cognitive complexities. Frittella (2019a) specifically addressed this phenomenon, demonstrating that decoding source language number words into different numerical formats requires extra conversion steps that engage distinct cognitive mechanisms depending on notation type.

Number notation, as a key factor in cognitive processing, influences how numbers are processed semantically, particularly in tasks involving magnitude conversion or numerical tasks. Research has shown that number notation engages distinct cognitive mechanisms depending on whether numbers are presented in Arabic digits or verbal number forms. Miceli (1990) and Kadosh et al. (2008) suggest that Arabic digits tend to lead to faster reaction times and fewer errors compared to verbal number words, as they activate faster and more efficient cognitive processes. Szűcs and Csépe (2004) corroborate this finding through an ERP study, demonstrating that Arabic digits are processed more quickly and with shorter reaction times than other notations. Furthermore, Ito and Hatta (2003) observed that Arabic digits led to faster brain responses compared to Japanese Kaji and Kana forms, supporting the idea that Arabic digits are processed more efficiently.

In the context of Chinese character numbers, evidence suggests that processing these characters is more cognitively demanding than processing Arabic digits, particularly in tasks such as number naming, magnitude selection, and basic arithmetic. Campbell et al. (1999, p. 12) specifically argued that Chinese character numbers, being “not used as often” compared to Arabic digits, demand more complex retrieval processes for magnitude judgments, thereby making magnitude information more difficult to activate during processing. Their experiments demonstrated that Arabic digits resulted in faster response times and fewer errors than Chinese character numbers. This work has been further developed through neuroimaging studies. W. Liu (2009) used functional Magnetic Resonance Imaging (fMRI) to show that brain activation during the processing of Chinese character numbers was stronger compared to Arabic digits, suggesting that mental processing of Chinese character numbers requires more cognitive resources due to their linguistic complexity. Cao et al. (2010a) provided additional neurocognitive evidence for notation-specific effects on semantic processing pathways for numerical information, while Cao et al. (2010b) reported longer reaction times for Chinese character numbers in a numeral parity judgment task compared to Arabic digits, further supporting the idea that different notations engage distinct cognitive mechanisms.

Despite these findings on notation effects in monolingual contexts, there remains a significant gap in understanding how number notation impacts cognitive processing in bilingual settings, particularly in interpreting tasks. While extensive research has examined simultaneous interpreting, much of the literature has focused primarily on auditory number perception, largely neglecting the impact of visual number presentation. Some studies have examined language-specific nuances, such as differences in magnitude systems (Korpal & Stachowiak-Szymczak, 2018; Cheung, 2023) and ASR accuracy (e.g., Pisani & Fantinuoli, 2021), —yet systematic investigation of the Chinese-English language pair, especially in sight translation tasks, remains rare. Sight translation is an underrepresented area in the literature, yet its immediacy and cognitive demands (Pöchhacker, 2022) make it particularly relevant for studying number interpreting. Unlike simultaneous or consecutive interpreting, sight translation requires interpreters to process written information visually while simultaneously producing oral output in another language, creating a unique cognitive challenge when processing numbers in different notation systems. The complexities of verbal number forms, such as number words or characters, remain largely unexplored in interpreting contexts.

With this imperative in mind, this study aims to address these gaps by examining the effect of number notation on the cognitive effort and performance of interpreters in Chinese to English sight translation. By using eye-tracking tools, this study aims to answer the following research questions: (1) How does number notation (Arabic digits vs. Chinese character numbers) affect interpreters’ cognitive effort, as measured by both global and local eye-tracking metrics and subjective evaluations? (2) To what extent does number notation influence the quality of sight translation both at the global (overall task performance) and the local level (number processing quality)?

## 3. Methods

This study employed a mixed-methods approach (Han, 2023), collecting eye-tracking data, performance measures, NASA-TLX ratings (Hart, 2006), and retrospective interviews to examine how number notation influences cognitive effort and performance in Chinese to English sight translation. This methodological triangulation provides complementary insights into interpreters’ cognitive processes, enriching quantitative findings through subjective data (Mellinger & Hanson, 2016). We used a counterbalanced within-subject design with number notation (Arabic digits vs. Chinese characters) as the independent variable, with each participant completing both conditions.

### 3.1. Participants

Eighteen trainee interpreters, all students enrolled in the Master of Translation and Interpreting (MTI) program in a Chinese university in 2020 participated in the experiment (see Table 1). All participants were native Chinese speakers with advanced English proficiency (certified with TEM-8, China’s authoritative English proficiency test for English majors) and had completed at least one semester of sight translation training. Their sight translation performance was comparable (scoring between 85–95/100 based on instructor evaluations), though most had limited or no professional interpreting experience. All participants had normal or corrected-to-normal vision, were familiar with the sight translation task, and provided informed consent before participating in the experiment.

### 3.2. Apparatus and Procedures

Participants completed two Chinese-to-English sight translation tasks using Tobii Pro Glasses 3 (120 Hz) in a quiet conference room. We selected wearable eye-tracking glasses based on Holmqvist et al. (2011, p. 93) who noted that head-mounted systems “allow the participants maximum mobility” in many real-life activities. This advantage over fixed systems enabled participants to maintain natural head movement and posture while performing visual processing tasks. Participants sat 3–4 m from a 150-inch screen displaying source texts as JPG images (1280 × 720 resolution), allowing clear and full view of the content without excessive upper body movements.

Task 1 involved interpreting text containing three large numbers presented in the “Arabic digit plus magnitude unit” form, while Task 2 used lowercase Chinese character numbers (see Table 2). To control for order effects, participants were randomly assigned to one of two sequence conditions. No time constraint or prior preparation was allowed for either task. Standard eye-tracking calibration preceded each task.

Following the tasks, participants completed an adapted 10-point NASA TLX questionnaire administered via the wenjuanxing platform (https://www.wjx.cn/, accessed on 28 August 2025) measuring six workload dimensions (Physical demand, Mental demand, Temporal demand, Performance, Effort, and Frustration). A semi-structured interview followed, focusing on perceived processing difficulty between number notations, effects on accuracy and fluency, and specific cognitive challenges encountered with each number format.

### 3.3. Stimulus

To ensure comparability and minimize confounding variables (Mellinger & Hanson, 2022), stimulus control in this study was meticulously implemented at three levels:

First, the numeric value of the three trigger numbers was matched in magnitude across both tasks (see Table 2), with only the notation format varying. Following Morrato (2011) who notes that large Chinese numbers exceeding millions are typically expressed in “Arabic digit plus magnitude unit” form to facilitate comprehension and processing, numbers in Task 1 were presented in this format, using units like “wan” (10,000) and “yi” (100,000,000). In Task 2, numbers were presented all in lowercase Chinese characters.

Second, text comparability was ensured by controlling source text attributes (see Table 2) in line with M. Liu and Chiu (2009). Both texts were of general content, with no subject-matter difficulties reported by participants. Lexical and syntactic parameters—including total word count, unrepeated words ratio, lexical density, incidence of difficult words and expressions, sentence count, characters per sentence, and number of complex sentences were matched. Unrepeated words, difficult words, and complex sentences parameters were calculated using the Chinese Readability Index Explorer (Sung et al., 2016).

Third, text difficulty was further validated by ten university interpreting instructors (bilingual Chinese L1, English L2; at least 5 years’ conference interpreting experience), who rated lexical complexity, syntactic complexity, information density, logic complexity, and comprehension difficulty. Paired *t*-tests (IBM SPSS 27) showed no significant difference between the two source texts across all dimensions (see Table 3), confirming stimulus comparability.

### 3.4. Data Processing

#### 3.4.1. Eye Movements Data Processing

**Figure 1:**
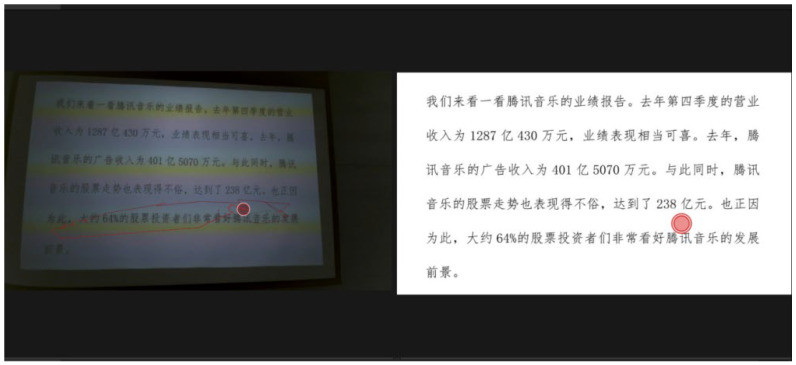
A screenshot of the assisted gaze-to-word mapping from Tobii Pro Lab.

Eye movements were processed using Tobii Pro Lab software Version 24.21.435 with the I-VT Filter (100ms minimum fixation threshold) based on Tobii Studio User’s Manual (2021). Eye-tracking data quality was carefully scrutinized (Hvelplund, 2014) under the following criteria: valid percentage of Gaze Samples (≥70%), Total Gaze Time (≥50% of task duration), and final Manual Check by the researchers to ensure that the eye-tracker effectively recorded participants’ eye movements on the screen. A 50% similarity threshold was applied for assisted gaze-to-word mapping (see Figure 1), to facilitate mapping of specific fixations to corresponding source text words (Carl & Kay, 2011). As is shown in Figure 1, the text displayed on the screen is the experimental material for the Arabic digit task. The red dots on the left and right sides of Figure 1 indicate a gaze-to-word mapping action, that is, on fixation of a participant (on the left side) is mapped to its corresponding location on the experimental stimulus (on the right side) for further analysis.

**Figure 2:**
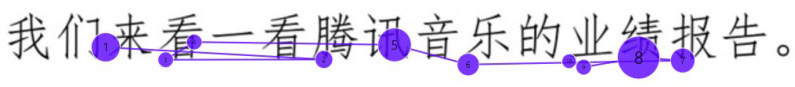
Fixations of one participant reading the Chinese source text in Task 1 is shown in Tobii Pro Lab. The numbers represent the sequence of fixations while the size of each dot represents duration of fixation.

**Figure 3:**
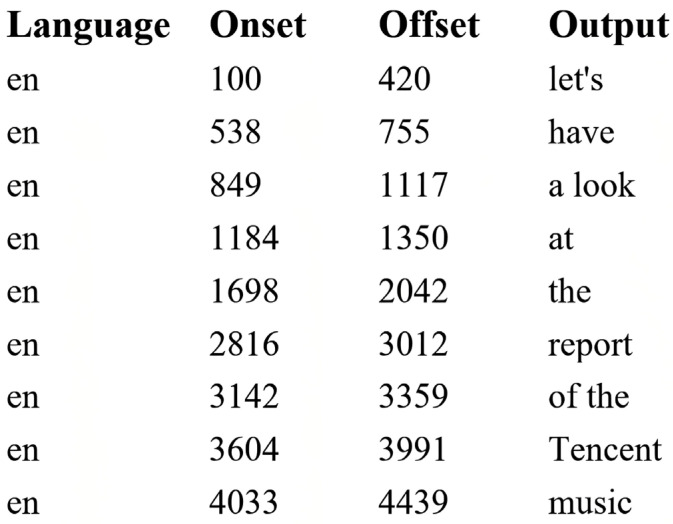
A screenshot of IBM Watson transcription output of one participant.

We analyzed eye movement metrics across two defined Areas of Interest (AOI)—the whole task area and trigger numbers—and analyzed various gaze metrics as detailed below. Eye-tracking metrics were computed at both global and local levels. At the global level, we analyzed Average Pupil Diameter (APD), Total Fixation Count (TFC), Total Fixation Duration (TFD), Total Saccade Count (TSC), Total Saccade Length (TSL), and Eye-Voice Span (IVS) across the whole task. At the local level, number-specific metrics included Average Pupil Diameter on Numbers (NAPD), Total Fixation Count on Numbers (NTFC), and Total Fixation Duration on Numbers (NTFD). Pupil diameter, measured as the average dilation across both eyes, serves as an indicator of mental effort—larger pupil diameters typically indicate greater mental effort, though dilation also reflects emotional states and arousal. Fixations—gaze events lasting at least 100 milliseconds—were identified (see Figure 2) using the I-VT fixation filter (Tobii Pro Lab User Manual, p. 65), ensuring meaningful distinction from rapid eye movements typically occurring in interpreting tasks. We analyzed both fixation count and duration. Saccade count and length were analyzed to capture participants’ visual processing traits, information processing efficiency, and attentional shift (Pluzyczka, 2013). Saccade length was calculated in pixels as the distance between successive fixation points using a custom program. Eye-voice span (IVS), a key indicator of visual-speech coordination (Chmiel & Lijewska, 2022; Chmiel, 2023), captures the efficiency of information processing (shorter spans) versus intensified cognitive effort (longer time lags). It was operationally calculated as the time interval between the first fixation on a specific source text segment (Eye onset) and the onset of its spoken interpretation (Voice onset). Voice onset timestamps were extracted from IBM Watson Speech-to-Text transcriptions at the token level (see Figure 3) and cross-verified with ELAN software. Mean IVS values derived from this procedure served as input for subsequent linear mixed-effects regression analysis.

#### 3.4.2. Sight Translation Quality Assessment

We analyzed sight translation quality through holistic assessment by experts and quantitative outputs analysis.

(1) Experts’ holistic assessment: Three expert raters (each with over ten years of interpreting experience) from the ten source text evaluators mentioned in Section 3.3 assessed the sight translation quality based on a ten-point scale including three parameters (Han & Riazi, 2017): information completeness (InfoCom), delivery fluency (DeliFlu), and target language quality (TLQual). Participant identities and task conditions were concealed during assessment, with mean scores across raters used for statistical analysis.
(2) Quantitative outputs analysis: We examined both global and number-specific performance indicators. At global level, we looked into target text lexical density (TLD, percentage of content words in the output), participants’ speech rate (SR, in syllables/second), target text production duration (TPD, in seconds), silent pause duration (SPD, in seconds), and filled pauses frequency (FPF, in counts). At local level, we analyzed number production duration (NPD, in seconds), filled pause frequency during number processing (NFPF, in counts), silent pause duration preceding number processing (NSPD, in seconds), number processing attempts (NPA1, in counts), and number processing acceptability (NPA2, percentage of acceptable outputs agreed by experts). The threshold for silent pause was 2 s based on empirical evidence that pauses of this duration or longer significantly impact fluency evaluations in interpreting (Macías, 2006). Filled pauses, “audible hesitations” (Macías, 2006, p. 27), take into account filler utterances such as “uh” and “um”, which do not carry concrete meaning but may negatively influence delivery fluency.

#### 3.4.3. NASA TLX and Retrospective Interview Analysis

The NASA TLX data were analyzed for each dimension separately to assess differences between tasks. For retrospective interviews, content analysis was conducted by identifying and categorizing participants’ responses according to the following themes: comparison of cognitive processing demand, performance differences, and specific challenges associated with number notation. The frequency and proportion of responses for each theme were calculated (e.g., percentage of participants reporting specific challenges with Chinese character numbers).

### 3.5. Statistical Analysis

We analyzed the data via linear mixed-effect regression (LMER) models using the lme4 package in R studio Version 4.3.1. We chose this statistical method for its merits of including both fixed and random effects in the model (Balling, 2008). Before performing the statistical analysis, we checked the distribution of the data in R and found that not all datasets followed a normal distribution. We applied Box-Cox transformations to data with skewed distributions (TFC, TSC, IVS, NTFC, NTFD, DeliFlu, SPD, FPF, TPD, NSPD, NFPF, NPD, and NPA1) and successfully made the data approximately normally distributed. After the necessary transformations, LMER models were built for each of the dependent variables in reading effort (APD, TFC, TFD, TSC, TSL, IVS, NAPD, NTFC, and NTFD), sight translation quality (InfoCom, DeliFlu, TLQual, TLD, NPA1, NPA2, SR, TPD, SPD, FPF, NPD, NFPF, and NSPD), and NASA TLX (Mentaldemand, Physicaldemand, Temporaldemand, Performance, Effort, and Frustration). For all the LMER models built, number notation types (NT) served as the fixed effect, and participants were entered as random effects to account for individual variability.

## 4. Results

This section reports key findings on how number notation affects participants’ cognitive effort and performance in Chinese-to-English sight translation. We present global cognitive effort metrics across notation conditions, followed by number-specific processing effects, performance quality differences, and participants’ subjective experiences from NASA-TLX assessments and interviews. The Section 5 will connect empirical findings with cognitive processing theories to explore the cognitive mechanisms underlying number processing during sight translation.

### 4.1. Reading Effort of Interpreters at Global and Local Level

Table 4 presents the descriptive statistics of eye movement metrics at both global and local level. Table 5 summarizes the results of LMER models testing the effect of number notation on these metrics.

#### 4.1.1. Reading Effort at Global Level: Task-Level Comparison

**Figure 4:**
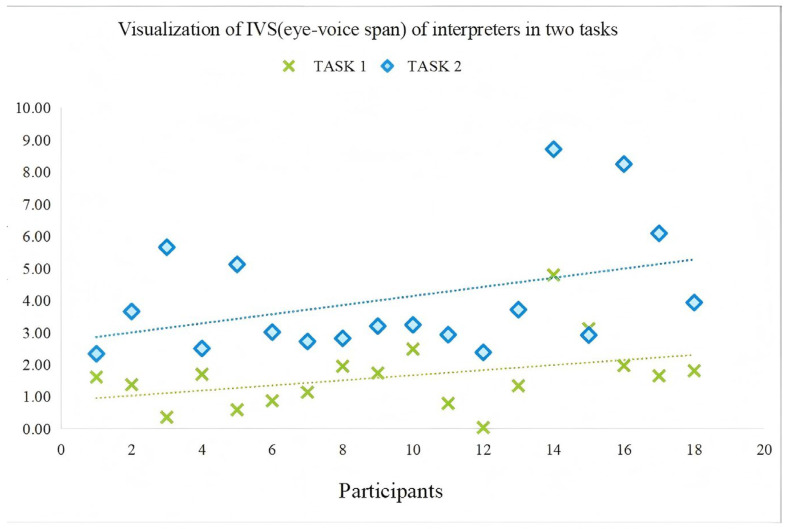
IVS of interpreters in the two tasks.

As is reflected in Table 4 and Table 5, several metrics revealed significant global task differences in reading effort. The total fixation count (TFC) was significantly higher (β = 0.130, SE = 0.054, *t* = 2.40, *p* = 0.03) in Task 2 (M = 250.1, SD = 84.80) compared to Task 1 (M = 188.1, SD = 56.08), indicating that participants made more fixations on the numbers when interpreting Chinese character numbers. Total fixation duration (TFD) also showed a significant increase (β = 15.037, SE = 1.475, *t* = 10.196, *p* < 0.001) in Task 2 (M = 57.76 s, SD = 21.87) versus Task 1 (M = 42.73, SD = 18.39), suggesting greater visual processing effort when interpreting texts with Chinese character numbers. Similarly, saccadic movements (TSC) were more frequent and extensive (β = 0.004, SE = 0.001, *t* = 3.81, *p* = 0.0006) in Task 2 (M = 446.78, SD = 227.99) than Task 1 (M = 363.39, SD = 183.84), reflecting higher cognitive effort when interpreting Chinese character numbers. The saccade length (TSL) also increased significantly (β = 10,768.47, SE = 1945.75, *t* = 5.534, *p* < 0.001) in Task 2 (M = 42,789.48, SD = 12,890.04) compared to Task 1 (M = 32,021.02, SD = 10,671.35), indicating more extensive visual search patterns when interpreting Chinese character numbers. While the average pupil diameter (APD) showed no statistically significant change (β = 0.047, SE = 0.028, *t* = 1.634, *p* = 0.121) between Task 1 (M = 3.22, SD = 0.25) and Task 2 (M = 3.27, SD = 0.22), the numerically higher APD values in Task 2 may suggest a tendency toward increased cognitive effort during Chinese character number processing. The eye-voice span (IVS, see Figure 4) was also significantly longer (β = 1.495, SE = 0.075, *t* = 19.75, *p* < 0.001) in Task 2 (M = 4.06, SD = 1.94) than in Task 1 (M = 1.63, SD = 1.09), further supporting the finding that Task 2 required participants to handle greater cognitive processing pressure. Collectively, these global eye-tracking metrics provide convergent evidence that processing Chinese character numbers during sight translation imposes substantially higher cognitive demands than processing Arabic numerals.

#### 4.1.2. Reading Effort at Local Level: Number Processing Comparison

Table 4 and Table 5 further show that participants exerted more reading effort processing Chinese character numbers compared to Arabic digits across the three eye-tracking metrics. The average pupil diameter processing numbers (NAPD) showed a significant increase (β = 0.252, SE = 0.032, *t* = 7.738, *p* < 0.001) in Task 2 (M = 3.69, SD = 0.25) compared to Task 1 (M = 3.43, SD = 0.27), suggesting that processing Chinese character numbers required greater cognitive effort. We also observed the significant influence of the number type (NT) on fixation count and fixation duration on numbers. For NTFC, more fixations (β = 0.9736, SE = 0.168, *t* = 5.77, *p* < 0.001) were found on Chinese character numbers (M = 77.78, SD = 53.10) than on Arabic digits (M = 41.28, SD = 34.17). Meanwhile, we also observed a significant increase (β = 0.731, SE = 0.165, *t* = 4.42, *p* < 0.001) in NTFD on the Chinese character numbers (M = 22.39, SD = 14.77) compared to Arabic digits (M = 12.23, SD = 10.05). These local-level metrics consistently demonstrate that Chinese character numbers demand significantly greater attentional resources, longer processing time, and higher cognitive effort compared to Arabic digits.

### 4.2. Sight Translation Quality at Global and Local Level

Having examined the reading effort metrics, we now turn to sight translation quality assessment. Table 6 presents the descriptive statistics of sight translation parameters at both global and local level. Table 7 summarizes the results of LMER models testing the effect of number notation on these metrics.

#### 4.2.1. Sight Translation Quality at Global Level: Task-Level Differences

As shown in Table 6 and Table 7, analysis of sight translation quality revealed significant differences between the two tasks. Expert holistic assessments showed that information completeness (InfoCom) was significantly lower (β = −9.004, SE = 1.65, *t* = −5.456, *p* < 0.001) in Task 2 (M = 69.73, SD = 11.45) compared to Task 1 (M = 78.74, SD = 9.87). Similarly, delivery fluency (DeliFlu) was significantly reduced (β = −556.90, SE = 106.03, *t* = −5.252, *p* < 0.001) in Task 2 (M = 69.66, SD = 8.66) versus Task 1 (M = 77.20, SD = 9.02), and target language quality (TLQual) was also significantly lower (β = −8.023, SE = 1.423, *t* = −5.64, *p* < 0.001) in Task 2 (M = 69.69, SD = 10.33) than in Task 1 (M = 77.70, SD = 8.97). These results suggest that Task 2 led to lower overall quality in sight translation.

In the quantitative analysis of sight translation outputs, TLD showed no significant task difference (β = 0.028, SE = 0.014, *t* = 2.013, *p* = 0.060) between Task 1 (M = 0.45, SD = 0.05) and Task 2 (M = 0.47, SD = 0.07), suggesting that the lexical complexity of the sight translation remained relatively consistent across both tasks. Similarly, the overall silent pause duration did not differ significantly between tasks (β = 0.047, SE = 0.085, *t* = 0.55, *p* = 0.589) when comparing Task 1 (M = 26.45, SD = 13.13) and Task 2 (M = 26.04, SD = 8.09), indicating that the time spent in silent pauses remained consistent across tasks. However, filled pause frequency showed a significant increase (β = 1.040, SE = 0.392, *t* = 2.65, *p* = 0.011) in Task 2 (M = 10.16, SD = 7.85) compared to Task 1 (M = 4.56, SD = 5.18), reflecting heightened cognitive effort, with participants possibly struggling to find appropriate translations for numbers in Task 2. Additionally, total production duration increased significantly (β = 20.078, SE = 3.313, *t* = 6.06, *p* < 0.001) in Task 2 (M = 99.19, SD = 27.90) versus Task 1 (M = 79.11, SD = 22.16), which suggests that participants required longer time to complete Task 2. Speech rate showed no significant difference (β = −0.007, SE = 0.060, *t* = −0.119, *p* = 0.906) between Task 1 (M = 2.10, SD = 0.33) and Task 2 (M = 2.09, SD = 0.46), indicating they managed to maintain a relatively consistent pace throughout both tasks. The significantly higher filled pause frequency and longer production time point to reduced delivery fluency and efficiency in the Chinese character number task. Overall, the results indicated reduced sight translation quality for Task 2 when compared to Task 1 at the global level in both experts’ assessments and quantitative analysis.

#### 4.2.2. Sight Translation Quality at Local Level: Number Processing Quality

According to Table 6 and Table 7, number processing metrics at the local level consistently showed significant task differences across the two conditions. We observed a significant increase (β = 0.432, SE = 0.194, *t* = 2.22, *p* = 0.03) in the silent pause duration before processing Chinese character numbers in Task 2 (M = 10.03, SD = 5.33) compared to Arabic digits in Task 1 (M = 5.64, SD = 4.29). This suggests that participants needed more time to prepare before processing numbers presented in Chinese character format. We also observed a significant increase (β = 0.981, SE = 0.279, *t* = 3.51, *p* = 0.002) in filler frequency during number processing in Task 2 (M = 8.66, SD = 6.64) compared to Task 1 (M = 3.44, SD = 3.97). This indicates that when processing Chinese character numbers, participants produced more fillers, suggesting a higher level of cognitive effort and uncertainty. A longer number processing duration (β = 0.933, SE = 0.417, *t* = 2.24, *p* = 0.033) was observed for Task 2 (M = 29.12, SD = 14.86) compared to Task 1 (M = 17.88, SD = 11.90). This suggests that participants took significantly more time to process numbers presented as Chinese characters. Participants also had significantly more (β = 0.736, SE = 0.276, *t* = 2.66, *p* = 0.014) number processing attempts in Task 2 (M = 6.67, SD = 2) compared to Task 1 with Arabic digits (M = 4.28, SD = 1.41), indicating that more attempts were needed when processing Chinese character numbers. Additionally, number processing acceptability showed a significant decrease (β = −2.5, SE = 0.429, *t* = −5.818, *p* < 0.001) in Task 2 (M = 46.6%, SD = 12.5%) compared to Task 1 (M = 71.6%, SD = 12.5%), suggesting lower acceptability when processing Chinese character numbers compared to Arabic digits, despite the significantly increased efforts and attempts.

### 4.3. NASA TLX and Retrospective Interview Results

Table 8 presents the descriptive statistics of NASA TLX rating metrics at both global and local level. Table 9 summarizes the results of LMER models testing the effect of number notation on these metrics.

According to Table 8 and Table 9, the NASA TLX analysis revealed significantly higher cognitive workload across all dimensions in Task 2 than Task 1. Mental Demand was substantially higher (β = 2.50, SE = 0.258, *t* = 9.659, *p* < 0.001) in Task 2 (M = 8.5, SD = 0.99) compared to Task 1 (M = 6, SD = 1.57). Physical Demand showed a similar significant increase (β = 2.055, SE = 0.205, *t* = 9.994, *p* < 0.001) in the Chinese character number task (M = 7.5, SD = 1.04) compared to the Arabic digit task (M = 5.44, SD = 1.25). A similar trend was observed for Temporal Demand (β = 2.166, SE = 0.345, *t* = 6.273, *p* < 0.001) in Task 2 (M = 7.89, SD = 1.08) versus Task 1 (M = 5.72, SD = 1.36), while Performance demonstrated a significant drop (β = −2.666, SE = 0.228, *t* = −11.66, *p* < 0.001) in Task 2 (M = 4.83, SD = 0.99) compared to Task 1 (M = 7.5, SD = 0.99), indicating worse self-perceived performance in the Chinese character number task. Significant increases were observed for both Effort (β = 2.944, SE = 0.221, *t* = 13.32, *p* < 0.001)in Task 2 (M = 8.55, SD = 1.09) versus Task 1 (M = 5.61, SD = 1.38), and Frustration (β = 3.0, SE = 0.412, *t* = 7.277, *p* < 0.001) in the Chinese character number task (M = 8.33, SD = 1.08) compared to the Arabic digit task (M = 5.33, SD = 1.57). These consistent patterns demonstrate that interpreting Chinese character numbers imposed substantially greater cognitive demands than processing Arabic digits, affecting both perceived performance and subjective workload.

Furthermore, insights from retrospective interviews provide additional evidence of the increased cognitive pressure associated with Chinese character numbers. 83.3% of the participants (15 out of 18) reported that interpreting Arabic numbers was less cognitively demanding compared to Chinese character numbers, despite both tasks being challenging. Additionally, 77.7% of the participants (14 out of 18) stated that their number processing accuracy and delivery fluency were significantly compromised in Task 2. A large majority (83.3%, 15 out of 18) of participants expressed substantial challenges with Chinese character numbers, specifically citing three key difficulties: unfamiliarity with numbers in character format, the disconnect between form and meaning in Chinese character numbers, and the inclination to mentally convert Chinese character numbers into Arabic digits before interpreting. These self-reported challenges align with the NASA TLX findings and help explain the decreased performance observed during Task 2, confirming the additional cognitive processing required for character-based numerical representations.

### 4.4. Key Findings

Collectively, the empirical evidence demonstrates that processing Chinese character numbers imposes substantially higher cognitive demands and yields compromised performance outcomes compared to Arabic digits in Chinese-to-English sight translation. Significantly elevated eye-tracking metrics (TFC/TFD/TSL/IVS/NAPD/NTFC/NTFD) revealed intensified visual processing effort for character-based numbers, corroborated by uniformly higher NASA-TLX ratings (Mental/Physical/Temporal Demand, Effort, Frustration). Concurrently, quality metrics exhibited systematic degradation: expert-rated parameters (InfoCom/DeliFlu/TLQual) declined significantly, while objective measures showed increased filled pauses (FPF/NFPF), prolonged processing duration (TPD/NPD), reduced acceptability (NPA2), and more correction attempts (NPA1). These convergent findings, supported by retrospective reports, establish that character-based numerical notation significantly increases cognitive load and reduces translation quality.

## 5. Discussion

This study investigated numerical notation effects in Chinese-English sight translation, revealing that notation format fundamentally alters both cognitive processing and performance outcomes. Our findings demonstrated that Chinese character numbers impose significantly greater cognitive demands and lead to compromised translation quality compared to Arabic numerals, even in the self-paced sight translation environment. Despite intensified number processing efforts, the number processing quality decreased significantly in the Chinese character number task. These results advance our understanding of interpreter cognition, numerical processing, and practical applications for interpreting education and technology development.

### 5.1. Greater Cognitive Effort at Global and Local Level in the Chinese Character Number Task

The first research question examined how number notation (Arabic vs. Chinese character numbers) affects interpreters’ cognitive effort, using a mixed-methods approach combining eye tracking, quality analysis, and subjective assessment. Our eye-tracking data reveal substantial notation-dependent differences across multiple indicators of cognitive effort. When processing Chinese character numbers, participants exhibited significantly more fixations (nearly doubled), longer fixation durations, and more extensive saccadic movements compared to Arabic numerals, indicating heightened visual attention and sustained cognitive engagement. Most notably, IVS showed a dramatic increase from 1.63 s in Task 1 to 4.06 s in Task 2 in processing time between perception and production. The increased Mental Demand and Frustration reported by participants in Task 2 further suggests that these numbers not only demand more cognitive resources but also create emotional strain due to their complexity and the additional cognitive steps required for their interpreting.

Our findings suggest that notation-independent semantic access of numbers, as proposed in Dehaene’s (1992) triple-code model, may not fully account for numerical processing across different formats. Instead, our results align with notation-dependent frameworks (Kadosh et al., 2008) and Campbell and Epp’s (2004) encoding-complex hypothesis, demonstrating that different notations engage distinct cognitive pathways with varying processing demands. The substantial eye-tracking differences we observed extend Campbell et al.’s (1999) and Cao et al.’s (2010a) monolingual findings that Chinese character numbers require more complex retrieval processes compared to Arabic digits, showing that these notation effects are not diminished but rather amplified in cross-linguistic contexts. As W. Liu (2009) demonstrated through fMRI evidence, Chinese character number processing activates more extensive prefrontal regions, suggesting heavier reliance on working memory resources—a finding our behavioral data corroborates through increased fixation patterns and processing delays.

The participants’ retrospective reports provide further insights into these processing differences, with 77% reporting that they mentally converted Chinese characters to Arabic numerals before processing—an additional transcoding step absent in the Arabic numeral task. This finding substantiates Frittella’s (2019a) assertion that number words necessitate an additional decoding step, which adds to overall task requirements and processing time. We refer to this extra cognitive operation “notation-induced transcoding cost”—a phenomenon where visual, perceptual, and semantic decoding of complex notations (e.g., Chinese characters) competes with core interpreting processes, consistent with Korpal and Stachowiak-Szymczak’s (2020) “processing bottlenecks.” The nearly 2.5 times increase in eye-voice span (IVS) for Chinese numbers suggests that participants must read ahead extensively to plan output, reflecting this bottleneck effect. This aligns with Pashler’s (1989) theory of attentional limitations during concurrent task processing—and fundamentally alters the temporal dynamics of sight translation (Chmiel & Lijewska, 2022).

The NASA-TLX subjective workload assessment further validates these objective measures, revealing significantly higher mental demand, physical demand, temporal demand, effort, and frustration when processing Chinese character numbers. Compared to previous studies on number interpreting in simultaneous interpreting (Desmet et al., 2018; Stachowiak-Szymczak & Korpal, 2019; Korpal & Stachowiak-Szymczak, 2020; Pisani & Fantinuoli, 2021), our findings reveal unique cognitive characteristics of the sight translation mode. Specifically, the continuous visibility of source text alters participants’ cognitive strategies, prompting them to make repeated processing attempts for all numbers rather than employing omission strategies. This suggests that interpreting cognitive processes dynamically adjust according to task modality (sight translation vs. simultaneous interpreting), potentially deepening our insights into the cognitive adaptability of interpreters.

### 5.2. Sight Translation Quality Declined at Global and Local Level in the Chinese Character Number Task Even with More Processing Attempts

The second research question explored how number notation influences the quality of sight translation. Our findings reveal a significant paradox: despite increased cognitive effort, participants achieved markedly lower performance with Chinese character numbers at both global and local levels.

Temporal analysis highlighted substantial processing difficulties with Chinese character numbers, manifested in longer total sight translation durations and nearly doubled silent pause durations preceding numbers (from 5.64 to 10.03 s). These extended pauses align with established research demonstrating longer reaction times for Chinese character processing due to linguistic-semantic complexity (Campbell et al., 1999; Cao et al., 2010a). The pattern of increased hesitation markers (filled pauses) corroborates Frittella’s (2019a) observation that participants employ such devices to “buy time” when confronting complex numerical processing tasks, reflecting heightened cognitive pressure (Plevoets & Defrancq, 2018).

Despite allocating significantly more cognitive resources to Chinese character numbers, participants achieved markedly lower performance outcomes, with number processing acceptability decreasing from 77.6% with Arabic numerals to 46.3% with Chinese characters. This creates an effort-quality paradox where increased processing effort corresponds with substantial performance degradation rather than improvement. While this number accuracy is higher than rates reported in previous studies in simultaneous interpreting mode such as Frittella’s (2019a) 35% and Korpal and Stachowiak-Szymczak’s (2020) 40%, likely due to the visibility of visual input and the self-paced nature of sight translation, the significant drop between notation conditions underscores the substantial impact of notation format on interpreting quality.

Notably, unlike previous studies documenting frequent number omissions in interpreting (Braun & Clarici, 1996; Mazza, 2001; Korpal & Stachowiak-Szymczak, 2020), our participants consistently attempted to process all numbers without resorting to omission strategies. This pattern differs from Plevoets and Defrancq’s (2018) finding that interpreters strategically omit numbers to manage cognitive load—a behavior observed even with visual assistance in some contexts (Yang, 2023). We identify this phenomenon as “sight translation trade-off”—where continuous visual access and no temporal pressure encourage persistent processing attempts rather than strategic omissions, potentially increasing overall cognitive burden through heightened monitoring demands. This phenomenon reveals a critical strategic limitation in our participants, who lacked the adaptive techniques that characterize expert performance under high cognitive load (Cheung, 2023). This behavior aligns precisely with Gile’s (2009, p. 181) observation in scripted simultaneous interpreting mode that, students “feel a strong temptation to rely on the text… because its content remains available in ‘solid’ print”, essentially treating the visual text as a cognitive safety net.

Interestingly, some participants employed approximation strategies despite the absence of time constraints—a counterintuitive finding that echoes Cheung’s (2023) explanation that approximation requires “fewer cognitive resources” than precise rendering. As participants reported in retrospective interviews, they primarily adopted this approach to enhance efficiency and minimize errors at smaller magnitudes when facing substantial cognitive demands. This adaptive behavior reflects the resource management strategies that emerge under cognitive strain (Gile, 2009), when interpreters tend to adopt processing shortcuts when specific task components consume excessive resources.

Our findings extend the literature on number processing in interpreting by revealing a critical interaction between notation format and task modality. While previous studies in simultaneous interpreting demonstrated positive correlations between visual processing effort and number interpreting accuracy (Desmet et al., 2018; Korpal & Stachowiak-Szymczak, 2020; Defrancq & Fantinuoli, 2021), our results suggest that in sight translation, this relationship is fundamentally altered by notation format. This challenges simplistic linear effort-performance relationships and supports more complex models of cognitive resource allocation. When specific processing tasks (such as Chinese character number decoding) consume excessive cognitive resources, performance on concurrent tasks (such as target language production) suffers, leading to overall quality deterioration.

Rather than simply reflecting insufficient attention, our findings suggest that number processing difficulties, particularly with complex notation systems, stem from fundamental limitations in working memory capacity and competing demands on central executive functions, as theorized by Pashler’s (1989) bottleneck theory. The notation-induced processing load alters participants’ strategic choices and risk tolerance, highlighting that notation format is not merely a superficial feature but a central determinant of task strategy, error patterns, and ultimate interpreting outcomes in sight translation modes.

### 5.3. Theoretical and Practical Implications

Based on the empirical findings presented above, our study provides empirical evidence of how number notation affects cognitive processing and performance in sight translation, revealing significant differences in processing demands between Chinese character numbers and Arabic digits. These findings align with Gile’s (2009) Effort Model, indicating that notation format can consume additional cognitive resources and impact overall interpreting performance, highlighting the importance of notation differences in cross-linguistic communication. By illuminating the complex interplay between number notation, cognitive processing, and interpreting performance, this study provides a foundation for both theoretical advancement and evidence-based practical innovation in interpreting research, education, and professional practice.

The sight translation trade-off we identified, where the continuous visibility of source text reduces omissions but potentially increases overall cognitive burden through heightened monitoring demands, challenges conventional assumptions about visual aid benefits in number interpreting. This modality-specific finding contributes to our understanding of the unique cognitive characteristics of different interpreting modes rather than assuming uniform cognitive processes across all settings. The effort-quality paradox observed in our study reveals how notation-specific processing demands create cognitive bottlenecks, as evidenced by 77% of participants reporting an additional mental step of transcoding Chinese characters into Arabic numerals—a specific cognitive load factor that significantly impacts resource allocation in interpreting tasks.

These theoretical insights translate into substantial practical applications for interpreter education. Training programs may consider including targeted notation-specific modules with particular emphasis on rapid transcoding between Chinese characters and Arabic digits. Exercises should be designed to automate the conversion process, gradually reducing the eye-voice span observed in our study. As suggested by Frittella (2019a), a three-stage “decoding-conversion-reconstruction” training model could be particularly effective, with specific focus on rapid decoding of Chinese character numbers. Additionally, simulation training should include various number notation formats and expression styles to enhance interpreters’ adaptability and psychological preparedness for diverse numerical challenges.

Our findings further reveal that notation-sensitive demands necessitate advanced computer-assisted interpreting (CAI) functionalities. While ASR technology improves numerical accuracy in simultaneous interpreting (Pisani & Fantinuoli, 2021), our data indicate these advantages are notation-dependent. Future CAI systems may consider integrating intelligent number detection and automatic conversion functions—resolving formatting inconsistencies flagged by J. Liu et al. (2024) and Defrancq and Fantinuoli (2021). Eye-tracking evidence further supports the development of adaptive interfaces that dynamically reformat numerals based on detected processing strain, creating responsive cognitive-load management tools. For multilingual communication stakeholders, our findings emphasize the importance of presentation format in multilingual settings. Conference organizers may consider prioritizing Arabic digits for statistical content, supplementing unavoidable Chinese characters with dual-format visual aids to reduce cognitive load and boost accuracy (Stachowiak-Szymczak & Korpal, 2019).

## 6. Conclusions

This study investigated the impact of number notation on cognitive effort and sight translation performance, demonstrating that Chinese character numbers impose significantly higher cognitive demands than Arabic digits, resulting in diminished performance despite increased processing attempts. Using eye-tracking methodology and quality assessment, we documented specific cognitive and performance effects of number notation, contributing to both theoretical understanding and practical applications in interpreting research.

Despite these contributions, we acknowledge several limitations in this study. First, our experimental design employed limited numerical stimuli (three numbers per task)—a methodological choice that prioritizes mechanism exploration over ecological validity, a trade-off noted by Mellinger and Hanson (2022) as common to balance data depth and breadth. While these constraints limit our ability to account for the full spectrum of number types, magnitudes, and contextual complexities encountered in real-world settings, controlled stimuli enabled precise eye-movement analysis and effective cognitive load management (Hvelplund, 2014), laying some groundwork for future, more ecologically valid studies. Secondly, our sample included only 18 MTI students and no professional interpreters, which limits generalizability across expertise levels. As Han (2023) points out, small samples—while typical in translation and interpreting experimental research—require “careful analysis, appropriate interpretation, and cautious generalization of empirical findings” (p. 246). This restricted participant pool prevents us from examining how professional experience moderates notation effects, highlighting an important direction for future investigation.

These preliminary findings suggest considerations that may be relevant to interpreter training and technological development. Interpreter education should prioritize automating cross-notation conversion skills, while cultivating adaptive strategies for notation switching under varying cognitive loads. As interpreting increasingly occurs in technologically-mediated environments (Defrancq & Fantinuoli, 2021), curricula must integrate training for collaborative engagement with CAI tools, emphasizing multi-source attention. These pedagogical refinements should be supported by notation-sensitive CAI functionalities that address specific cognitive challenges identified in our research, including real-time digit conversion, enhanced numerical visualization, and adaptive interfaces that mitigate cognitive bottlenecks during number-dense interpreting tasks. Our findings also suggest that standardizing numerical notation in multilingual settings—through preferential use of Arabic digits or dual-notation formats—could substantially reduce cognitive burden for both interpreters and diverse audience members.

Future research should address the limitations of our study through several approaches. Including experienced professional interpreters in future samples could clarify how expertise shapes notation processing and reveal adaptive strategies developed through practice. Methodologically, combining eye-tracking with neuroimaging would offer more nuanced insights into the neural mechanisms underlying notation processing, while employing more diverse number types and realistic contexts would enhance ecological validity. Cross-modal and cross-linguistic studies could further map the generalizability of notation effects across different interpreting modes (e.g., simultaneous interpreting with ASR, traditional consecutive interpreting) and language pairs with varied notation systems. Finally, experimental evaluation of notation-aware CAI tools and longitudinal studies of specialized training outcomes would strengthen the link between cognitive research and professional practice. As interpreting practices evolve with new technologies, research examining the cognitive and linguistic dimensions of notation processing may contribute to our understanding of effective multilingual communication strategies.

## Figures and Tables

**Table 1:** Participants Profile.

Participants	Age	Language	Gender
18 MTI students	Range (23–28) M = 24.6 (SD = 1.99)	Chinese (L1), English (L2)	14 females, 4 males

**Table 2:** Quantitative comparison of the two source texts.

Description	Task 1	Task 2
Independent variable Three trigger numbers in Chinese (and their English translations)	1287亿430万元 (128 billion, 704 million, and 300 thousand Yuan) 401亿5070万元 (40 billion, 150 million, and 700 thousand Yuan) 238亿元 (23 billion and 800 million Yuan)	六千五百四十三亿六百四十万元 (654 billion, 306 million, and 400 thousand Yuan) 九百零五亿四千九百三十万元 (90 billion, 549 million, and 300 thousand Yuan) 七百八十九亿元 (78 billion and 900 million Yuan)
Lexical parameters		
Word count (character)	120	120
Unrepeated words ratio	0.63	0.64
Lexical density	0.75	0.74
Difficult words	13	15
Syntactic parameters		
Number of sentences	6	6
Characters per sentence	20	20
Complex sentences	1	1
Expert assessment	M = 5.52	M = 5.46
Text complexity (10)	SD = 0.89	SD = 1.03

**Table 3:** 10 bilingual experts’ mean rating of source text complexity.

Category	Task 1 M (SD)	Task 2 M (SD)	Paired *t*-Test
Lexical complexity (out of 10)	5.5 (0.70)	5 (0.73)	*p* = 0.23
Syntactic complexity (out of 10)	4.8 (0.63)	4.9 (0.73)	*p* = 0.75
Information density (out of 10)	7 (0.94)	7.3 (0.95)	*p* = 0.49
Logic complexity (out of 10)	5.5 (0.71)	5 (0.82)	*p* = 0.16
Comprehension difficulty (out of 10)	4.8 (1.03)	5.1 (0.86)	*p* = 0.49

**Table 4:** Reading effort at global and local level: descriptive statistics.

Gaze Category	Items	Task 1 M (SD)	Task 2 M (SD)
Global	APD (millimeter)	3.22 (0.25)	3.27 (0.22)
	TFC (count)	188.1 (56.08)	250.1 (84.80)
	TFD (seconds)	42.73 (18.39)	57.76 (21.87)
	TSC (count)	363.39 (183.84)	446.78 (227.99)
	TSL (pixel)	32,021.02 (10,671.35)	42,789.48 (12,890.04)
	IVS (seconds)	1.63 (1.09)	4.06 (1.94)
Local	NAPD (millimeter)	3.43 (0.27)	3.69 (0.25)
	NTFC (count)	41.28 (34.17)	77.78 (53.10)
	NTFD (seconds)	12.23 (10.05)	22.39 (14.77)

**Table 5:** Reading effort at global and local level: LMER results. (The * sign represents results of statistical significance).

Gaze	Items	Term	Estimate	SE	*t*	*p*	Random Effect (Variance)	Random Effect (Std. Dev.)
Global	APD	Intercept	3.223	0.054	58.937	<0.001	0.046342	0.2153
		T2	0.047	0.028	1.634	=0.121		
	TFC	Intercept	3.675	0.130	28.20	<0.001	0.13675	0.05726
		T2	0.130	0.054	2.40	=0.03 *		
	TFD	Intercept	42.728	4.763	8.971	<0.001	388.75	19.717
		T2	15.037	1.475	10.196	<0.001 *		
	TSC	Intercept	1.467	0.059	24.59	<0.001	5.32 × 10^−5^	0.007290
		T2	0.004	0.001	3.81	=0.0006 *		
	TSL	Intercept	32,021.02	2789.02	11.481	<0.001	115,941,932	10,293
		T2	10,768.47	1945.75	5.534	<0.001 *		
	IVS	Intercept	0.390	0.106	3.68	<0.001	0.2351	0.4849
		T2	1.495	0.075	19.75	<0.001 *		
Local	NAPD	Intercept	3.433	0.061	56.041	<0.001	0.057955	0.2407
		T2	0.252	0.032	7.738	<0.001 *		
	NTFC	Intercept	4.174	0.079	52.44	<0.001	0.9317	0.9647
		T2	0.973	0.168	5.77	<0.001 *		
	NTFD	Intercept	2.260	0.138	16.32	<0.001	0.5087	0.7134
		T2	0.731	0.165	4.42	<0.001 *		

**Table 6:** Sight translation quality at global and local level: descriptive statistics.

Gaze Category	Items	Task 1 M (SD)	Task 2 M (SD)
Global	InfoCom (100)	78.74 (9.87)	69.73 (11.45)
	DeliFlu (100)	77.20 (9.02)	69.66 (8.66)
	TLQual (100)	77.70 (8.97)	69.69 (10.33)
	TLD (percentage)	0.45 (0.05)	0.47 (0.07)
	SR (syllables/second)	2.10 (0.33)	2.09 (0.46)
	TPD (seconds)	79.11 (22.16)	99.19 (27.90)
	SPD (seconds)	26.45 (13.13)	26.04 (8.09)
	FPF (counts)	4.56 (5.18)	10.16 (7.85)
Local	NPD (seconds)	17.88 (11.90)	29.12 (14.86)
	NSPD (seconds)	5.64 (4.29)	10.03 (5.33)
	NFPF (counts)	3.44 (3.97)	8.66 (6.64)
	NPA1 (count)	4.28 (1.41)	6.67 (2)
	NPA2 (percentage)	71.6% (12.5%)	46.6% (12.5%)

**Table 7:** Sight translation quality at global and local level: LMER results. (The * sign represents results of statistical significance).

Gaze	Items	Term	Estimate	SE	*t*	*p*	Random Effect (Variance)	Random Effect (Std. Dev.)
Global	InfoCom	Intercept	78.743	2.52	31.251	<0.001	89.76	9.474
		T2	−9.004	1.65	−5.456	<0.001 *		
	DeliFlu	Intercept	3018.24	147.3	20.490	<0.001	289,442	537.9
		T2	−556.90	106.03	−5.252	<0.001 *		
	TLQual	Intercept	77.707	2.281	34.07	<0.001	75.04	8.683
		T2	−8.023	1.423	−5.64	<0.001 *		
	TLD	Intercept	0.447	0.014	30.590	<0.001	0.002063	0.045
		T2	0.028	0.014	2.013	=0.060		
	SR	Intercept	2.103	0.094	22.245	<0.001	0.12784	0.357
		T2	−0.007	0.060	−0.119	=0.906		
	TPD	Intercept	79.109	5.940	13.32	<0.001	23.157	9.939
		T2	20.078	3.313	6.06	<0.001 *		
	SPD	Intercept	3.272	0.147	22.22	<0.001	0.1178	0.343
		T2	0.047	0.085	0.55	=0.589		
	FPF	Intercept	1.428	0.147	9.69	<0.001	0.9141	0.956
		T2	1.040	0.392	2.65	=0.011 *		
Local	NSPD	Intercept	1.292	0.139	9.26	<0.001	0.882	0.2970
		T2	0.432	0.194	2.22	=0.03 *		
	NFPF	Intercept	1.208	0.234	5.16	<0.001	0.6816	0.8252
		T2	0.981	0.279	3.51	=0.002 *		
	NPD	Intercept	3.509	0.352	9.95	<0.001	0.7875	0.8873
		T2	0.933	0.417	2.24	=0.033 *		
	NPA1	Intercept	1.771	0.244	7.24	<0.001	0.1999	0.4460
		T2	0.736	0.276	2.66	=0.014 *		
	NPA2	Intercept	7.166	0.324	22.076	<0.001	0.2353	0.4851
		T2	−2.5	0.429	−5.818	<0.001 *		

**Table 8:** NASA TLX rating: descriptive statistics.

Items	Task 1 M (SD)	Task 2 M (SD)
Mental demand	6 (1.57)	8.5 (0.99)
Physical demand	5.44 (1.25)	7.5 (1.04)
Temporal demand	5.72 (1.36)	7.89 (1.08)
Performance	7.5 (0.99)	4.83 (0.99)
Effort	5.61 (1.38)	8.55 (1.09)
Frustration	5.33 (1.57)	8.33 (1.08)

**Table 9:** NASA TLX rating: LMER results. (The * sign represents results of statistical significance).

Items	Term	Estimate	SE	*t*	*p*	Random Effect (Variance)	Random Effect (Std. Dev.)
Mental demand	Intercept	6.00	0.309	19.407	<0.001	1.1176	1.0572
	T2	2.50	0.258	9.659	<0.001 *		
Physical demand	Intercept	5.444	0.271	20.091	<0.001	0.9412	0.9701
	T2	2.055	0.205	9.994	<0.001 *		
Temporal demand	Intercept	5.722	0.289	19.747	<0.001	0.4379	0.6617
	T2	2.166	0.345	6.273	<0.001 *		
Performance	Intercept	7.500	0.232	32.30	<0.001	0.5000	0.7071
	T2	−2.66	0.228	−11.66	<0.001 *		
Effort	Intercept	5.611	0.29	19.12	<0.001	1.1111	1.054
	T2	2.944	0.221	13.32	<0.001 *		
Frustration	Intercept	5.333	0.318	16.756	<0.001	0.2941	0.5423
	T2	3.000	0.412	7.277	<0.001 *		

## Data Availability

The datasets analysed during the current study are available on Figshare via the web-link: https://figshare.com/s/38a4b5a48cd4a5e52efc.

## Abbreviations

The following abbreviations are used in this manuscript:

AOI	area of interest
APD	Average Pupil Diameter
ASR	automatic speech recognition
CAI	computer-assisted interpreting
DeliFlu	Delivery fluency
fMRI	functional Magnetic Resonance Imaging
FPF	Filled pauses frequency
InfoCom	Information completeness
IVS	eye-voice span
LMER	Linear mixed-effects regression
L1	first language
L2	second language
MTI	Master of Translation and Interpreting
NAPD	Average Pupil Diameter on Numbers
NASA TLX	NASA Task Load Index
NFPF	Filled pause frequency during number processing
NPA1	Number processing attempts
NPA2	Number processing acceptability
NPD	Number production duration
NSPD	Silent pause duration preceding number processing
NT	Number notation types
NTFC	Total Fixation Count on Numbers
NTFD	Total Fixation Duration on Numbers
SPD	Silent pause duration
TFC	Total Fixation Count
TFD	Total Fixation Duration
TLD	Target text lexical density
TLQual	Target language quality
TPD	Target text production duration
TSC	Total Saccade Count
TSL	Total Saccade Length
